# A Cost Modelling System for Recycling Carbon Fiber-Reinforced Composites

**DOI:** 10.3390/polym13234208

**Published:** 2021-12-01

**Authors:** Essam Shehab, Arshyn Meiirbekov, Akniyet Amantayeva, Aidar Suleimen, Serik Tokbolat, Shoaib Sarfraz

**Affiliations:** 1 Mechanical and Aerospace Engineering Department, School of Engineering and Digital Sciences, Nazarbayev University, Nur-Sultan 010000, Kazakhstan; arshyn.meiirbekov@nu.edu.kz (A.M.); akniyet.amantayeva@nu.edu.kz (A.A.); aidar.suleimen@nu.edu.kz (A.S.); 2 School of Architecture, Design and the Built Environment, Nottingham Trent University, Nottingham NG1 4FQ, UK; serik.tokbolat@ntu.ac.uk; 3 Design, Manufacturing and Production Engineering Section, Department of Mechanical and Production Engineering, Aarhus University, 8000 Aarhus, Denmark; ssarfraz@mpe.au.dk

**Keywords:** carbon fiber-reinforced composites’ recycling processes, cost modelling, KBS

## Abstract

Cost-effective and environmentally responsible ways of carbon fiber-reinforced composite (CFRP) recycling are increasingly important, owing to the rapidly increasing use of these materials in many industries such as the aerospace, automotive and energy sectors. Product designers need to consider the costs associated with manufacturing and the end-of-life stage of such materials to make informed decisions. They also need to understand the current methods of composite recycling and disposal and their impact on the end-of-life costs. A comprehensive literature review indicated that there is no such tool to estimate CFRP recycling costs without any prior knowledge and expertise. Therefore, this research paper proposed a novel knowledge-based system for the cost modelling of recycling CFRP that does not require in-depth knowledge from a user. A prototype of a cost estimation system has been developed based on existing CFRP recycling techniques such as mechanical recycling, pyrolysis, fluidized bed, and supercritical water. The proposed system has the ability to select the appropriate recycling techniques based on a user’s needs with the help of an optimization module based on the Technique for Order of Preference by Similarity to Ideal Solution (TOPSIS). Estimating recycling costs has taken into consideration various factors such as different material types in different industries, transportation, and dismantling costs. The developed system can be employed to support early-stage designers and decision-making stakeholders in terms of understanding and predicting recycling costs easily and quickly.

## 1. Introduction

Carbon fiber-reinforced polymer matrix composites (CFRPs) are being rapidly adopted among emerging composite materials across various industries such as in aircraft and wind turbine blade manufacturing as well as in the transportation sector [1]. The global market capacity for CFRP was estimated to be approximately USD 5 billion in 2019 and it was expected to grow by 10.6% annually, reaching around USD 8 billion in 2024 [2]. In terms of the worldwide production of CFRP, it is estimated to reach almost 200 kt by 2022, whereas the amount produced in 2018 was 128 kt [3]. The reason behind such a relatively high demand is related to the superior properties of composite materials such as higher strength, lower weight (25 to 75% reduction in weight), and corrosion resistance compared to conventional materials such as steel and aluminum. As a result, using CFRPs enables energy saving and reducing carbon emissions associated with the life cycle of the final products. For example, recycling a kilogram of carbon fiber with a chemical method consumes 38 MJ of energy, whereas the production of virgin carbon fiber requires 5–15 times more [3]. Moreover, composites form more than half of the share of materials used in the manufacturing of the new generation aircrafts such as the Boeing 747 Dreamliner and Airbus A350 [4]. This is gradually leading to a situation where composites are becoming more attractive in industries where steel and aluminum are currently predominant.

Such progress in the widespread usage of CFRPs has been slightly slowed across various industries, including aerospace, automotive, and wind energy sectors, which have been affected by the ongoing COVID-19 pandemic. Among others, new challenges were reported to take place across the CERPs supply chain such as the absence of raw material, financial hardship, and a lack of line workers in the production process. The global restrictions on international travelling have led to a decrease in the level of air travel, which in turn decreased the demand for new aircraft and, consequently, for carbon fiber (CF) and CFRP materials. Nevertheless, there is still potential waste that is coming from existing products which requires urgent consideration. For example, Boeing 777s and Airbus A350 aircraft, with more than 50% of the materials being carbon fibers, are estimated to reach their end-of-life stage in the next decades [5]. At present, nearly 400 commercial aircraft reach their end-of-life stage every year in the world, generating around 1000 metric tons of carbon fiber waste [6]. Moreover, 30% of carbon fiber ends up as manufacturing waste resulting from cutting or trimming operations during product manufacturing [7]. Therefore, challenges related to the disposal of end-of-life material and manufacturing waste still exist.

The main challenge associated with the massive production of CFRPs is their recycling. The conventional ways of disposing waste such as landfilling and incineration cause a negative environmental impact and are no longer preferred under the European Union’s Waste Framework Directive(Directive 2008/98/EC) [1]. Moreover, environmental legislation in some countries has demanded companies to recycle up to 85% of all weight of end-of-life products and recover 10% of it as energy starting from 2015 [8]. However, the current recycling rate is still low (no more than 2000 tons per year) due to several technical factors [9]. One issue is related to the complexity of the material structure, which is composed of mixed cross-linked thermosets that cannot be remelted. Table 1 demonstrates that thermosets dominate in the current market compared to other types of carbon fiber composites.

Another reason is the diversity of mixtures of composites that do not allow using standardized processes for the collection and sorting of waste. Finally, composite materials contain cores and coatings which require man force to be separated for recycling [11].

Along with the technical challenges, cost predication also tends to hinder the process of growth of composite waste’s recycling rate. For instance, there are recycling methods that are not commercially viable due to their high dependence on energy consumption. Moreover, the recycled composites are often considered to be of lower quality in contrast to virgin composites; thus, the area of application is restricted to internal aircraft structures, for instance. Finally, composite waste recycling plants tend to be located far from the suppliers of the waste, which in turn, requires transportation cost supply and supply chain-related performance to be taken into consideration [12].

Boeing has established good practices of recycling carbon fiber waste by recycling up to 100% of its CFRP waste in cooperation with the company ELG Carbon Fibre based in the UK. The partnership resulted in training employees and arranging recycling processes on 11 manufacturing sites [13]. Other carbon fiber (CF) recycling companies include Carbon Conversions (Lake City, SC, USA), HADEG Recycling GmbH (Stade, Germany), ELG Carbon Fibre Ltd. (Bilston, UK), and Takayasu Co., Ltd. (Gifu, Japan) [7]. Moreover, CF manufacturers tend to express interest in recycling as producing recycled CF consumes ten times less energy than virgin material. The energy and cost reduction are the strong drivers for recycling CF on the market. For example, recycled CF (rCF) costs around USD 18–25 per kg, whereas virgin CF (vCF) is valued at USD 33–66 per kg [14].

The production of vCF is expensive but also energy-intensive (energetic cost is 183–286 MJ/kg) [15]. Recycled CF can decrease costs by 70% and energetic costs by almost 98% [16]. Saved energy from using rCF is equal to the annual electricity use of 175,000 homes [16].

The increased application of carbon fiber-reinforced composites across various industries along with rising environmental concerns requires developing financially viable and effective recycling techniques. Different recycling techniques have been developed over the last twenty years. The most prominent techniques are mechanical, thermal (pyrolysis), and chemical (solvolysis) processes [15]. In the case of mechanical recycling, fiber and matrix are separated by shredding and then followed by grinding, resulting in flakes, powder and fibrous fractions [17]. In the case of thermal recycling techniques, among which are pyrolysis and fluidized bed processes, heat is used to decompose matrices and convert them into gases, tar, and char [18,19]. Pyrolysis is a process used at an industrial scale by most of the recycling companies; for example, ELG Carbon Fibre operates with a capacity of 2000 tons/year [20]. Finally, the solvolysis technique adopts chemical reactions in different organic liquids at high-pressure or supercritical conditions to break the matrix. Other techniques such as electrochemical and biotechnological techniques have also been developed but they are less advanced compared to others [21]. 

At present, more cost-effective ways of recycling CFRP are being developed. However, only a few of them offer proper business models for commercialization or integration into current waste management systems. Despite the increasing attention to recycling CFRPs, there is a gap in terms of developing cost modelling and its software tools for recycling carbon fiber composites. 

Limited studies have examined the financial performance of the CFRP recycling process. Li et al. conducted a life-cycle cost analysis of mechanical recycling and the further application of recycled carbon fibers [22]. According to the study, the low recovery rates from the process and low value for rCF were not enough to cover the costs of processing the waste. Meng et al., in turn, performed a financial analysis on the viability of the fluidized bed process for recycling CF and further applications in the automotive industry [23]. The study provided a comprehensive financial model and sensitivity analysis in order to find out that the carbon fibers can be recycled at the price of USD 5 per kg, which is equal to 15% of vCFs. A study by Vo Dong et al. [24] developed an economic and environmental model of different waste disposal routes for assessing their performance. Except for the traditional disposal routes such as landfilling and incineration, the recycling options were mechanical recycling, pyrolysis, microwave pyrolysis, and solvolysis in supercritical water. The study reported profound knowledge about various financial aspects of the considered recycling techniques. Xu et al. [25] modelled the costs of end-of-life automotive components for different recycling options. The reuse (remanufacturing) options of the crankshaft and composite material oil pan have been selected for the study, which involves reconditioning procedures. The developed model provided a cost structure with a prominent example of an activity-based cost estimation. Hagnell and Akermo [18] proposed the recyclate value model which modelled the potential of the closed-loop application of fiber-reinforced materials. The modelling tool evaluates the cost of recycled fiber with the connection to mechanical properties degraded after recycling. The study reported that 50% of cost reductions can be achieved with the comparable level of mechanical properties using recycled fiber for certain applications. Lefeuvre et al. [26] modelled a pyrolysis plant using Aspen Hysys v8.6 software to estimate the environmental savings and financial implications of recycling CFRPs. The results showed that USD 4.3 mln of capital investments is necessary to pilot a pyrolysis plant with 1500 tons/year capacity. La Rosa et al. conducted a life-cycle cost analysis of recycling CF thermosets using solvolysis which included only materials, transport, labor, and energy costs [27]. According to the authors, the open-loop recycling (resulting in shredded CF) costs were EUR 288 per 35.5 kg, whereas the same amount of material for closed-loop recycling (long CF equivalent to vCFs) accounted for EUR 2.91. Hoefer developed a framework for economic decisions in wind turbine blade disposal [28]. The developed framework has inputs such as blade parameters, selling price, landfilling tax, etc., which allows for choosing between options such as remanufacturing, landfilling, and processing blades to sell a recyclate. 

The literature review indicated that no effort was made in developing a knowledge-based cost modelling tool to support selecting the recycling option of carbon fiber composites. Moreover, the research in this field is limited by industry type, recycling process and supply chain considerations. In other words, there is no record of a system that considers several waste sources (manufacturing, industrial), recycling processes and the whole recycling supply chain including waste transportation and dismantling when calculating the final cost of recycling CFRP. There is a lack of cost models that consider several factors simultaneously. Such a model could be helpful in understanding the recycling cost drivers and understanding the influence of recycling plant parameters and desired quality on the cost of recyclates for each recycling method. The cost estimation of recycling, particularly at the conceptual design stage, is a critical and, at the same time, difficult task. This research work aimed to develop a cost estimating model and its knowledge-based prototype software tool for different techniques of recycling CFRPs. The system has the capability of selecting suitable recycling processes that meet the user requirements.

## 2. Development of a Cost Model for Recycling CFRPs

CFRP recycling stages and their associated cost elements such as disassembly, transportation, capital investments (e.g., construction of a plant), and operating costs were taken into consideration to provide a fundamental assessment of the economic viability of recycling carbon fiber composites. The cost model was developed for recycling techniques to be assessed in terms of their capital costs (CAPEX) such as equipment/construction, and operational costs (OPEX) such as utilities, labor, depreciation, overhead, etc. The standard 10 years’ project lifespan of a project was assessed for economic viability. Taxes and subsidies were not considered in the analysis by assigning a zero (0) value as the tax legislation varies from state to state. However, these inputs could be altered by a user. The economic indicators that allow assessing the break-even price for selling rCFs and utilities cost are represented at the end of this section. Additionally, the sensitivity analysis was performed to provide an insight into the uncertainty of input data such as recovery rate and annual capacity, which could significantly affect the results.

The contribution of variable and fixed costs were determined by performing classical estimates and comparisons with similar research works [23,24]. The cost-related input data are given in Table 2. A 10-year depreciation period with a linear pattern was assumed. The capital investment costs were determined using the rule of six-tenths, according to which the designed capacity data can be adjusted to another intended capacity [29]. The operational costs including utilities and energy costs were obtained from the literature [30,31]. The labor cost was extracted from the official data of Eurostat (40-h working week with a wage of EUR 31.4 per hour) [32]. For all recycling techniques, it was assumed that the operating labor consists of four people, and the same assumption was made by Vo Dong et al. [24]. These parameters can be adjusted by a user. 

In terms of the economic indicators, the approach used by Vo Dong et al. [24] was adopted and the following assumptions were made:

1. Utilities cost per 1 kg of waste (UC). This represents the sum of all utility expenses for the chosen method.
2. An average unit cost per 1 kg of waste recovered (UCW). For this purpose, a break-even value at zero net present value (*NPV*) is calculated. A discount rate of 10% is assumed for calculations.
3. The main parameter assessed is the average unit cost per 1 kg of fiber recovered (UCF). This parameter allows determining the break-even price of selling the recovered product.

The latter two parameters are referring to costs with two different perspectives: the unit cost of recovered waste (UCW) could be useful for waste handlers, whereas the unit cost per fiber recovered (UCF) could reflect the final cost of recycled fibers. 

The formula for *NPV* could be found in Equation (1) [24]:

(1) $$NPV=-C_{tc}+\overset{10}{\underset{i=1}{\sum }}\frac{-Annual\;cost*\left(1-a\right)+D}{{\left(1+\propto \right)}^{i}}$$

where,

$-C_{tc}$—total capital costs
a—tax rate (in this study, it is assumed to be zero (0))
D—depreciation (linear)
∝—discount rate (10%)

### 2.1. Cost Elements

There are three cost elements that were considered in this study, namely, capital cost for the recycling factory, transportation cost and disassembly cost. Capital cost focused on four recycling techniques which are pyrolysis, mechanical recycling (grinding), the fluidized bed process, and solvolysis in supercritical water. These processes had been considered both by the research community and industry and offer tangible results. 

This work is focused on the recovery pathways of carbon fiber. The choice of these methods is based on the literature review results and current practices predominant in the CFRP recycling industry. The material assessed in the study is assumed to have 65% of CF content except for the material considered in the supercritical water related study, in which authors have tested material with 50% fiber content [33].

Pyrolysis is one of the most developed and recognized methods in the industry with a good recovery rate of fibers’ mechanical properties despite the high energy requirements. According to the study by Zhang et al. [34], the technology readiness level (TRL) of pyrolysis for CFRP has a value of eight (8) that corresponds to the “system/subsystem” development level. On the other hand, the solvolysis process performing the best in terms of recovery rates of CF properties corresponds to a TRL of 4 (“technology development” stage), most likely due to issues related to achieving positive profit values. Mechanical recycling is the simplest method for the recycling of composite materials. The material in this method is processed using shredders and millers. This technology results in the considerable deterioration of the mechanical properties of rCF. This tends to limit their capability to be utilized in high-value parts [5]. Finally, the fluidized bed process is one of the emerging methods and is characterized by relatively high tolerance levels to contaminated materials [35].

Although the recovery rates in the mentioned technologies do not reach 100%, the retention rate of the properties of recovered carbon fibers is promising. In this study, a 100% fiber recovery rate was assumed for the following processes: pyrolysis, fluidized bed process, and solvolysis in supercritical water [24]. The recovery rate for the grinding process is assumed to be 40%, which was adopted from the study of Li et al. [22]. 

#### 2.1.1. Capital Cost

The capital costs estimation is adopted from the literature by combining the rule of six-tenths and chemical engineering plant cost indices (CEPCI) [36,37]. According to the rule of six-tenths, the approximate cost of a new facility can be estimated based on the historical data of the previous facility at a different facility. After that, a CEPCI is used to adjust the cost data for the current period of estimation. The estimates were carried out in the year 2020 with the latest known CEPCI in the year 2019 [38]. The formula used for calculating the adjusted capital cost per design is shown below:

(2) $$C_{d}=C_{r}{\left(\frac{d}{r}\right)}^{0.6}\frac{I_{2019}}{I_{i}}$$

where,

$C_{d}$—capital costs of a plant for a capacity ton per year
$C_{r}$—reference capital costs of a plant from the literature
r—indicated capacity in the literature
$I_{2019}$—CEPCI index in 2019
$I_{i}$—CEPCI index for the year of a reference plant

Table 3 summarizes the capital costs used in the study with the adjusted CEPCI indices used for the cost model.

#### 2.1.2. Pyrolysis

Pyrolysis is a thermal method that performs the decomposition of a matrix in the absence of oxygen at temperatures varying between 400 and 700 °C [35]. The method offers a number of advantages over other alternatives that recover fibers with retained mechanical properties; however, it still has its drawbacks. The decomposition process leaves char on the surface of the material which in turn negatively affects the performance characteristics of fiber [5]. There were recent developments achieved that allowed for the removal of the char by applying carbon dioxide and water vapor, opening new horizons for the more advanced application of the technology in the industry [43]. It is important to mention that the cost model for pyrolysis in this work does not include the char-removal step but only the main spending on the process.

The capital costs were adapted from Vo Dong et al. [24], i.e., estimates of EUR 10,000,000 for a capacity of 50,000 tons of waste recovered annually. The capital costs were adjusted according to the CEPCI. The energy consumption rate of 30 MJ/kg is taken as a reference value from the study of Witik et al. [44]. However, some studies report the energy consumption rates being as low as 2.8 MJ/kg [45]. The energy from the accompanying products of the process was not considered. 

#### 2.1.3. Mechanical Recycling

Mechanical recycling is the most mature method of recycling composite materials with several steps of decreasing recyclate size [34]. In this method, the material is cut into pieces 50–100 mm in size and fed into a shredder. The pieces are then transformed into particles 10 mm to 50 μm in size [46]. The resultant recyclate material can be categorized by fiber content and fraction. Palmer et al. conducted a study on the classification of the recyclate [47].

The capital costs are adopted from the ERCOM plant with a capacity of 4000 tons per year with a shredder cost of EUR 200,000 [40]. The hammer mills are presented in the market as having a price of approximately a quarter of the shredder’s cost with a capacity of 25–40 t/hour [48]. The plant was established in 1990 and was shut down in 2004 due to economic reasons [49]. The capital cost values were adjusted accordingly from the year 1990 using the CEPCI 358 [41]. The energy consumption levels during grinding are adopted from the equation derived by Howarth et al. [50] with the approximate consumption of 0.27 MJ/kg at the capacity of 150 kg/hour.

(3) $$E=11.15\times (Q_{m}{}^{-0.76})$$

where,

*E*—energy consumption in MJ/kg
$Q_{m}$—capacity at kg/hour

#### 2.1.4. Fluidized Bed Process

The fluidized bed process was developed to recover high-grade glass and carbon fibers under moderate temperatures. In the process of recycling, the scraps with a reduced size of up to 25 mms are fluidized with a hot stream of air in a bed at temperatures varying between 450 and 550 °C [46]. Although the initial studies on the fluidized bed process reported losses in terms of tensile strength, Zheng et al. [51] reported an over 95% recovery rate of fibers after using the fluidized bed technique. The distinctive feature of the fluidized bed process is its capability to treat materials with contaminants.

In general, the fluidized bed process requires capital investments of EUR 4.1 million for the capacity of 1000 tons/year [23]. The estimate was adjusted by the latest known CEPCI for the year 2019. The total energy consumed by the fluidized bed process has been estimated to be 6 MJ/kg [52].

#### 2.1.5. Supercritical Water

Solvolysis in supercritical water is a process in which the polymer matrix is decomposed for recovering CFs. The method provides the highest recovery rate with no or minimal decrease (1–2%) reported in tensile strengths compared with original fibers [53,54]. However, the method is not commercialized widely due to issues in terms of achieving profit. It was reported that substantial capital investments are needed in terms of equipment that can withstand excess pressures and temperature during the process [34,55].

According to Knight [33], for the solvolysis in the supercritical water method, EUR 4.9 million in capital investments for a plant working at a capacity of 150 kg/h are needed. Additionally, for 1 kg of composite material waste (50% wt), this recycling method requires 3.47 kWh of electricity, 19.75 kWh (1.90 m^3^) of natural gas, 96 kg of cooling water and 4.6 kg of pure water. The prices are indicated in Table 4.

#### 2.1.6. Transportation Cost

As the model considered in this study is based on a hypothetical composite material treatment, specific locations of theoretical plants are not defined. This creates uncertainty. Nevertheless, the transportation distance cost assumed in this study was adopted from Li et al. as EUR 0.047 per km [22]. 

#### 2.1.7. Disassembly Cost

Dismantling costs for the automotive industry were assumed to be EUR 1.53 per kg based on the data obtained from Li et al. [22]. For the wind turbine industry, disassembly costs were extrapolated from different cost values pertinent to various wind turbine sizes of the Suncor Energy Project and were assumed to be equal to EUR 0.42 per kg [56]. For the aerospace industry, dismantling costs were obtained from publicly available sources. The average value of EUR 0.54 per kg is assumed based on the calculation of the dismantling costs of Boeing 747 reported by Cacciottolo [57]. It is important to note that these values are extremely vague and were used as indicative values; thus, the user is advised to calculate the disassembly costs for each case and enter the system.

## 3. Proposed Modeling Approach of Carbon Fiber (CF) Recycling Costs 

### 3.1. The Overall Architecture of the Proposed System

**Figure 1:**
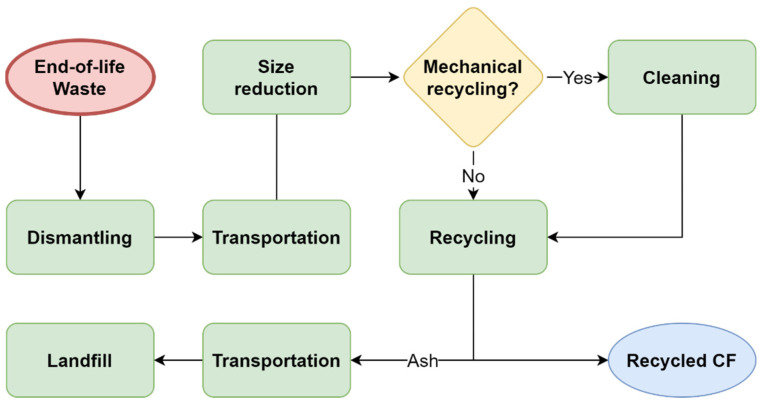
Recycling process flow of carbon fiber campsites.

The CFRP recycling process flow is shown in Figure 1 which indicates the required steps starting from the end-of-life waste to the resulting recycled CF. The costs are incurred at all stages, and therefore, are added to the total cost estimation. For example, dismantling, transportation, and size reduction costs exist in all types of recycling processes. However, only the mechanical recycling method requires cleaning which increases the cost of the process. Moreover, size reduction of large-scale materials, such as wind turbine blades, might be necessary before transportation. It is worth mentioning that treating residues (for example, ash) is not considered after recycling CF in the total cost calculation due to their negligible values. 

**Figure 2:**
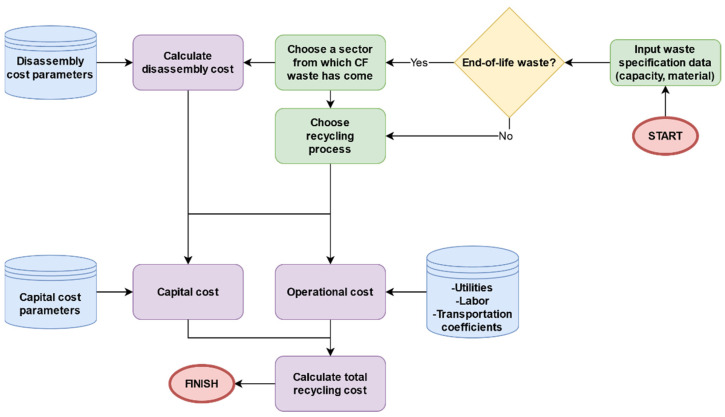
Overall structure of the developed system.

Figure 2 illustrates the overall structure of the proposed software system for the cost estimation of recycling carbon fiber campsites. The cost of recycling consists of dismantling costs, capital costs, and operational costs. Each cost element is estimated according to the user input parameters and predefined coefficients allocated to each cost element (e.g., labor, transportation cost). The system consists of two main modules: (1) a knowledge-based system (KBS), which is composed of if-then rules to select appropriate recycling process, and (2) a database that stores all the data entries by the user along with the waste recycling specification data. 

The proposed rule-based system selects appropriate recycling processes and estimates capital, operational, disassembly and transportation costs required for CFRP recycling. For example, the algorithm for the selection of the recycling process according to predetermined rules is given in Table 5.

**Figure 3:**
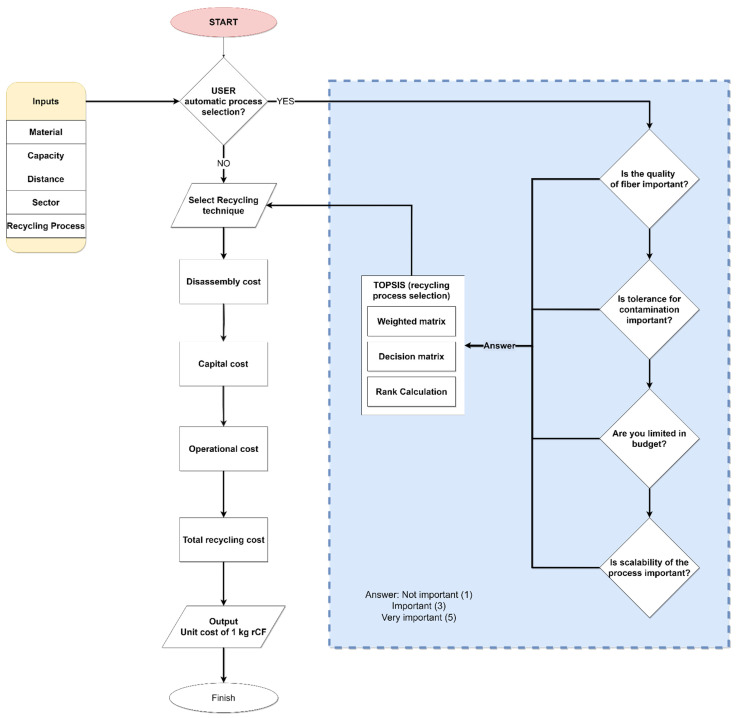
System scenario for the total recycling cost estimation process.

The system scenario of the proposed cost analysis process is shown in Figure 3. The system prompts a user to enter all the necessary characteristics of the waste material to be recycled such as the waste type and its weight. Such data is stored in the project database. The user selects the desired recycling process or chooses the automatic selection feature which suggests the recycling method according to the user’s previously specified criteria. The waste characteristics are the main input to the cost estimation module. The selection of the cost estimation and recycling method requires continuous interaction between different modules such as the waste specification database and CFRP waste recycling process knowledge base. The knowledge base module consists of a set of rules for selecting an appropriate recycling process by utilizing the Technique of Ranking Preferences by Similarity to the Ideal Solution (TOPSIS). The TOPSIS method finds the alternative that is closest to the ideal solution and farthest from the most negative ideal solution [58].

### 3.2. Optimization Module

To propose the appropriate recycling process for selection, a multicriteria decision-making analysis was conducted according to the user’s potential criteria/requirements. The Technique of Ranking Preferences by Similarity to the Ideal Solution (TOPSIS) was used to solve the multicriteria decision-making (MDCM) problem. The TOPSIS is a convenient and simple technique that can take into account a significant number of alternatives. The purpose of this method is to calculate the distance to the ideal solution, which is adjusted by the user’s preferences [58].

#### Criteria Quantification

Four criteria are available for assisting the user in the process of selecting the desired recycling method. The values are assigned ranging from 1–5 corresponding to the importance of the criterion from the least to the highest. Table 6 shows the quantified values for the assessed methods. The values are assigned based on the information obtained from the literature review.

The chosen criteria are further analyzed following the steps below:

1. The construction of the comparison matrix, which is illustrated in Table 5. The matrix constructed is based on the four (4) recycling methods and respective criteria. According to Lee and Chang [59], the columns represent criteria and rows represent the respective methods.
2. The matrix is normalized using Equation (4) [58,59,60]:
  (4) $$\bar{X_{ij}}=\frac{X_{ij}}{\sqrt{\sum_{i=1}^{n}X_{ij}^{2}}}$$
  where, $\bar{X_{ij}}$—normalized value; $X_{ij}$—real value in the matrix.
3. The normalized matrix is adjusted by weights incurred from user inputs and calculated using the Equation (5) [58,59,60]
  (5) $$V_{ij}={\bar{X}}_{ij}\times W_{j}$$
4. Ideal negative and ideal positive solutions are determined using Equations (6) and (7) [58,59,60]
  (6) $$A^{+}=\left\{\max\;V_{ij}\right\}=\left\{\mathrm{The}\;\mathrm{maximum}\;\mathrm{value}\;\mathrm{of}\;\mathrm{each}\;\mathrm{column}\;\mathrm{in}\;V_{ij}\right\}\;$$
  (7) $$A^{-}=\left\{\min\;V_{ij}\right\}=\left\{\mathrm{The}\;\mathrm{minimum}\;\mathrm{value}\;\mathrm{of}\;\mathrm{each}\;\mathrm{column}\;\mathrm{in}\;V_{ij}\right\}\;$$
  where, $A^{+}$—positive ideal solution; $A^{-}$—ideal negative solution.
5. Euclidean distances are calculated from ideal positive and ideal negative solutions using Equations (8) and (9) [58,59,60]
  (8) $$S_{i}^{+}=\sqrt{\sum_{j=1}^{m}{\left(V_{ij}-V_{j}{}^{+}\right)}^{2}}$$
  (9) $$S_{i}^{-}=\sqrt{\sum_{j=1}^{m}{\left(V_{ij}-V_{j}{}^{-}\right)}^{2}}$$
6. Performance score Pi is calculated using the formula provided below [58,59,60]
  (10) $$P_{i}=\frac{S_{i}^{+}}{S_{i}^{+}+S_{i}^{-}}$$

### 3.3. System Implementation and Validation

**Figure 4:**
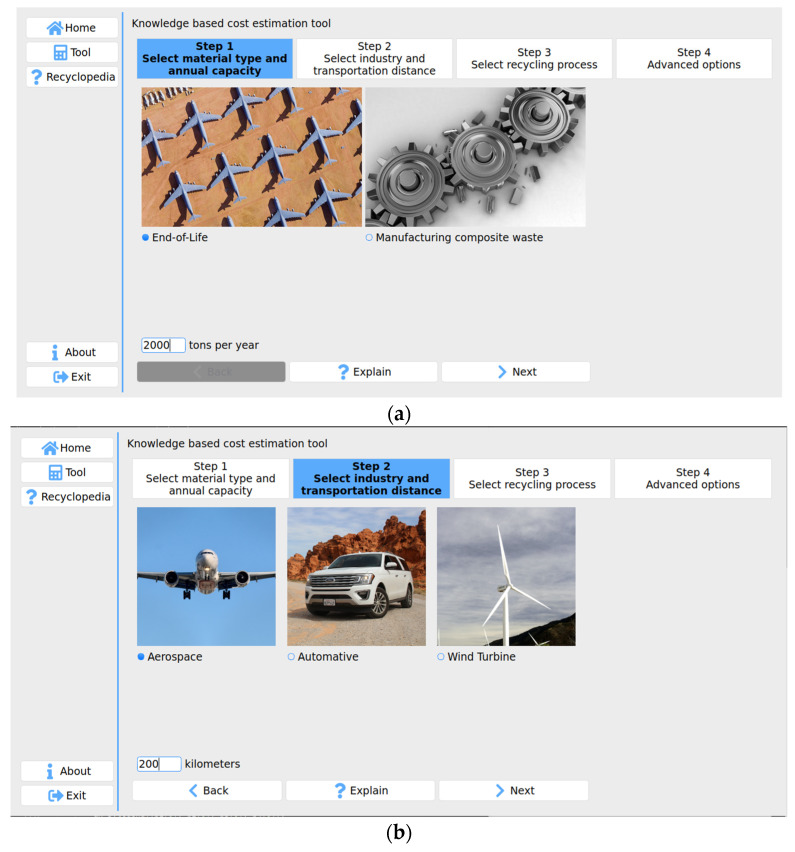

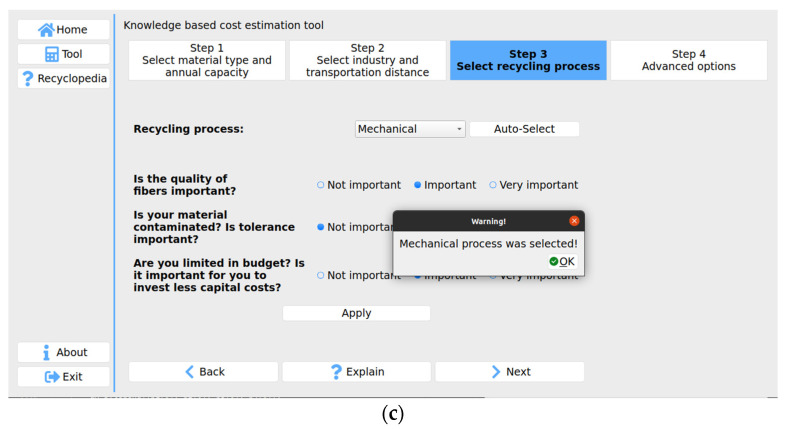
(**a**) Screenshot of material type selection and annual capacity; (**b**) industry sector selection; (**c**) recycling process recommendation.

A prototype software-based system was developed to implement the cost modeling methodology using Python 3 and PyQt5. Python is a powerful object-oriented programming language that supports big data and complex mathematics [61]. It also provides the necessary tools to build knowledge-based systems. PyQt5 is a Python library used for building graphical user interfaces (GUI). It allows the user interface to be written in a coded format that will be transformed into an automatic layout [62]. The software runs on any PC under Windows OS and macOS and is designed to be menu-driven so that there are fewer manual input entries. A user-friendly interface has been developed to allow users to use the software efficiently. In the system, the user is asked to answer questions and enter parameters in four steps which are represented in Figure 4a–c. The user has to specify a material type and annual capacity. He or she should select the industry sector for waste generation and input transportation distance between end-of-life products or manufacturing waste and recycling factory. The system has the capability of allowing the user to choose a recycling process or recommend a recycling process based on the user inputs and TOPSIS or based on predefined parameters.

**Figure 5:**
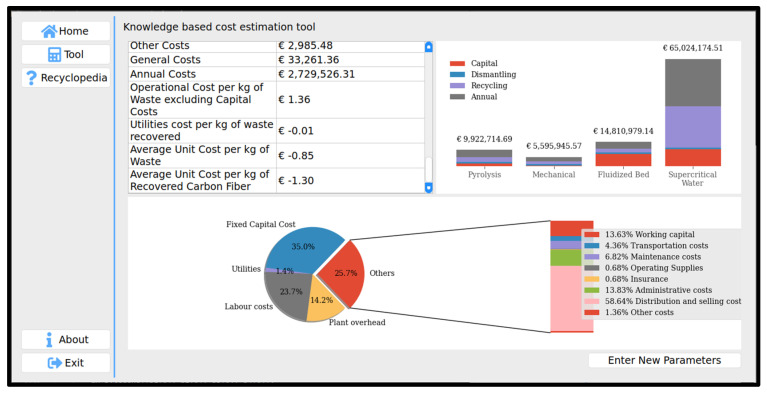
Cost estimation results.

Figure 5 shows the cost estimation results generated by the developed system. The system output illustrates the total cost of four recycling processes. The system enables the user to change the input parameters and compare results.

#### 3.3.1. System Validation: Case Study

Public data from a leading recycling carbon fiber composite company were employed in the developed system. ELG Carbon Fibre, targeted as a case study, is a recycling company based in the UK with 60 employees and a 4000 m^2^ warehouse. The pyrolysis furnace installed at this company has a capacity of recovering 1500 tons of carbon fiber per year. The process contains three steps used for carbon fiber recovery and further production: (1) the mechanical shredding of laminates and prepregs; (2) a pyrolysis process; and (3) milling/non-woven mat production. At the current capacity of the supply chain of 1300 tons, it is noted that the recycled products cost about EUR 10–20 per kg, whereas the costs of virgin fiber products vary between EUR 30 and 40 per kg [63]. To validate the system, the closest parameters to the aforementioned conditions were input into the system. 

Table 7 shows the values of input parameters provided to the system and the unit cost per waste and per recovered CF obtained as a result. The unit cost recovered of fiber is EUR 6 per kg, which can be used as a raw material for the further processing and creation of rCF products. The difference between the indicated value and the system output can be explained by several factors. Firstly, it is important to note that the production of woven mats is not considered in this study, as the scope of the system is concerned only by the recycling process itself. Secondly, the estimation of recycling costs does not include taxes, which vary in different countries. Finally, it is clear that the price of recycled products ranging between EUR 10 and 20 per kg also includes profit margins, which allow the continuous operation of these plants, whereas EUR 6 per kg of the unit cost of rCF is a reasonable estimate for the main operation of fiber reclamation.

#### 3.3.2. Sensitivity Analysis

Sensitivity analysis is an approach that shows how much a single uncertainty parameter could affect the output value. In this study, the system output is analyzed by changing the input parameters such as annual capacity, recycling process, and carbon fiber recovery rate. It should be noted that the sensitivity analysis does not consider the effect of factors acting simultaneously on the cost estimate, but only separately. Therefore, there is no probability distribution, and the sensitivity analysis is carried out based on single values.

**Figure 6:**
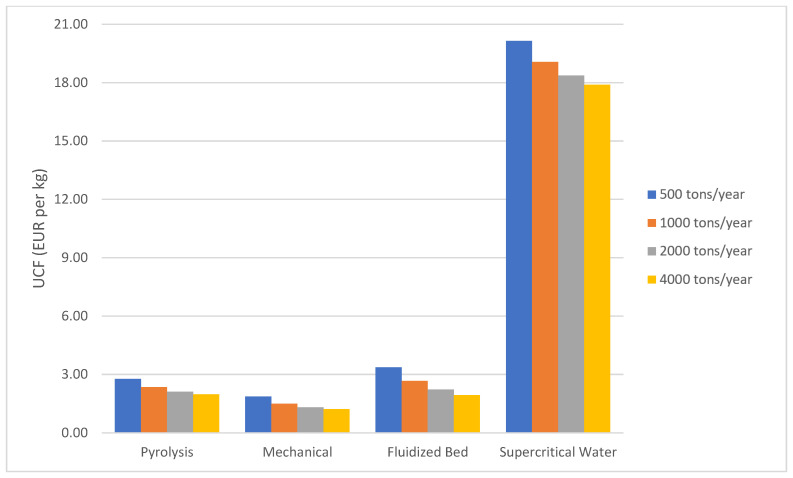
Unit cost per mass of recovered carbon fiber at 100% recovery rate.

Figure 6 shows the average unit cost per mass of recovered carbon fiber (UCF) for four different recycling processes and four different annual capacities. It assumed a 100% carbon fiber recovery rate and shows that as the annual capacity increases, the unit cost of the recovered fiber decreases. The increase in annual recycling capacity has a significant effect on the UCF of all processes except for supercritical water. The difference in recycling costs between 500 and 4000 tons for the fluidized bed process, mechanical recycling, and pyrolysis represented 43%, 35%, and 29%, respectively. However, supercritical water had only an 11% decrease in the UCF under the same terms. 

From the analysis, it can be stated that the UCF from pyrolysis, mechanical recycling, and fluidized bed process at the shown capacities can successfully compete with the manufacturing costs of cheap lignin-based carbon fiber (EUR 5.3 per kg) [64]. Solvolysis in supercritical water resulted in the highest UCF, which can be explained by large initial investments and utility costs. However, the process has the highest retention rate of properties amongst others and has potential in high-value applications. The estimated cost in the analysis (EUR 17.9–20.1 per kg) is still comparable with the reported cost of manufacturing carbon fibers from the polyacrylonitrile (PAN) precursor of non-aerospace grade (EUR 18.3 per kg), which still makes the process economically viable [65].

**Figure 7:**
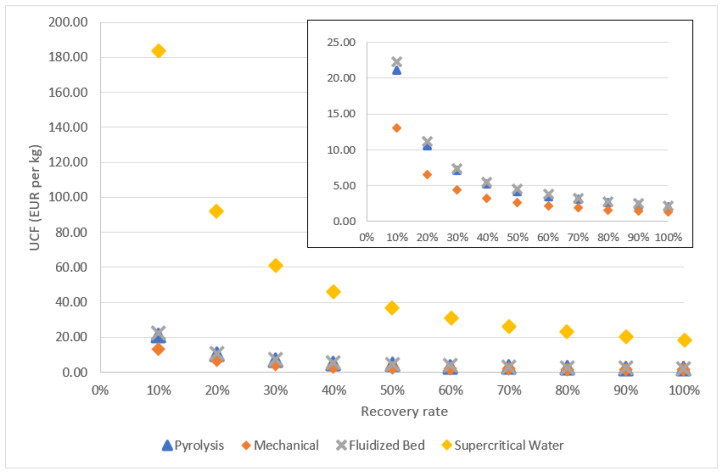
Unit cost per mass of recovered carbon fiber at 2000 tons/year and at different recovery rates.

In Figure 7, the average unit cost per mass of recovered carbon fiber is presented against the recovery rate of carbon fiber. The recovery rate varied from 10% to 100%. Logically, increasing the recovery rate reduces the average recycling cost of recovered carbon fiber. Supercritical water has the highest UCF regardless of recovery rate compared to other methods. Thermal methods including pyrolysis and the fluidized bed process result in similar UCF with increasing recovery rates; however, the UCF from pyrolysis is still lower compared to the fluidized bed process at any recovery rate. At the chosen capacity, these methods must have a recovery rate higher than 40% to be competitive compared to the cost of carbon fiber made of the polyacrylonitrile (PAN) precursor. Mechanical recycling has the lowest UCF amongst others; although, at a 10% recovery rate, the UCF of the process (EUR 13.1 per kg) becomes less attractive compared to thermal methods at recovery rates higher than 10%. It is also noted that the UCF from mechanical recycling with the recovery rate adopted in this study (40%) (EUR 3.3 per kg) is still higher than compared to costs yielded from pyrolysis and the fluidized bed process at their recovery rate. 

## 4. Conclusions

Estimating the end-of-life treatment cost is vitally important for early-stage designers, manufacturers and industry members in order to optimize the product and budget. Currently, the recycling industry spends a lot of resources on cost modelling of such new systems, especially in their early stage of development. Cost estimation requires expert knowledge in the recycling technical and business processes, which is difficult to gain owing to a lack of data and information available in the field. Therefore, a knowledge-based system for the cost prediction of various carbon fiber recycling techniques has been proposed. The recycling techniques such as mechanical recycling, pyrolysis, the fluidized bed process, and solvolysis in supercritical water were considered in this study. The prototype software was developed with a user-friendly interface, knowledge-based system, and optimization tool for selecting the suitable recycling process for different scenarios. The developed methodology estimates the total costs of CFRP recycling according to specified inputs. It also allows for taking into account exogenous factors such as transportation costs, disassembly costs, industry, and material differences. Moreover, the optimization module based on the TOPSIS assists the user in choosing the recycling process based on the most important criteria such as capital investments, scalability, the quality of fibers, and contamination tolerance. Additionally, the sensitivity analysis revealed that all methods are positively affected by the economy of scales, though the supercritical water technique is affected the least amongst them. The brief comparison with the prices of virgin carbon fibers revealed that all methods are cost-competitive, though supercritical water requires an almost 100% recovery rate to be economically viable. The findings of this research work could provide insights for both decision-makers namely, waste handlers and waste recyclers.

However, the focus of the work was on estimating recycling costs and recommending suitable recycling process based on the user’s needs. Further research efforts are required to examine possible applications of rCFs and estimating the costs of manufacturing products from rCFs. Moreover, investments should be made to develop the data management approach in order to feed the system with appropriate and up-to-date information from the industry and further automate the cost estimation process. In addition, the impact of uncertainty factors on the cost estimation of recycling CFRPs requires further investigation. The cost drivers in the recycling processes might have a variation and alter the final cost of recycling CFRPs depending on the country’s energy balance, for example. Hence, the development of the cost uncertainty estimation framework and incorporating it into the system could be a future research area. This will allow for estimating the range of recycling costs and conducting statistical analysis with confidence intervals which will improve the reliability of the estimates provided by the system.

## Figures and Tables

**Table 1:** Distribution of the global CFRP market by matrix material [10].

Matrix Type		Market Size (bln USD)	Market Share	
Hybrid		1.2		5.2%
Metal Matrix		0.82		3.5%
Ceramic matrix		4.65		20.1%
Polymer matrix	Thermoplastic	4.72	28.8%	71.2%
	Thermoset	11.37	69.0%	
	Hybrid and Others	0.36	2.2%	

**Table 2:** Cost input data model.

Cost Type	Estimate Calculation
Fixed capital costs (Cfc)	Capital investments
Working capital costs, (Cwc)	10% of Cfc
Total capital costs	A sum of fixed and working capital costs
Dismantling costs	Based on a sector type
Recycling costs	
Direct	
Utilities	Based on a technique type
Labor costs	4 operating staff members
Transportation costs	Based on a chosen distance
Maintenance costs	5% of Cfc
Operating supplies	10% of Maintenance costs
Indirect	
Plant overheads	60% of Operating labor
Insurance	0.5% of Cfc
Depreciation, D	10% linear
General costs	
Administrative costs	25% of plant overhead costs
Other costs	1% of Cfc
Distribution and selling costs	1% of all expenses

**Table 3:** Capital investment and used CEPCI indices for this study.

Technique	Capital Investment According to Literature	CEPCI Year	CEPCI Index	Adjusted Capital Costs in the Model
Pyrolysis	EUR 10,000,000 for a capacity of avg. 50,000 tons per year [24]	2012	585 [39]	EUR 10,384,615 for a capacity of avg. 50,000 tons per year
Mechanical	EUR 200,000 for a capacity of 4000 tons per year (only shredder) [40]	1990	350 [41]	EUR 452,514 for a capacity of 4000 tons per year (a hammer miller included)
Fluidized bed	EUR 4,100,000 for a capacity of 1000 tons per year [23]	2015	558 [23]	EUR 4,483,058 for a capacity of 1000 tons per year
Supercritical Water	EUR 5,770,000 for a capacity of 150 kg per hour [33]	2013	567 [42]	EUR 6,178,874 for a capacity of 150 kg per hour

Further subsections will cover the data about cost drivers utilized in the cost model for each process.

**Table 4:** Utility expenses.

Utility Type	Cost per Unit
Electricity	EUR 0.0801 per kWh [30]
Natural gas	EUR 0.0308 per kWh (EUR 0.32 per m^3^) [31]
Cooling water	EUR 12.58 per 1000 kg [33]
Pure water	EUR 2.08 per kg [33]

**Table 5:** The algorithm for the selection of the recycling process.

***IF***	*(Quality of recovered fibers is not important)*	*AND*
	*(Scalability of the process is very important)*	*AND*
	*(Tolerance for contamination is very important)*	*AND*
	*(Capital cost amount is not important)*	
***THEN***	*(The recycling process is pyrolysis)*	
***IF***	*(Quality of recovered fibers is not important)*	*AND*
	*(Scalability of the process is very important)*	*AND*
	*(Tolerance for contamination is very important)*	*AND*
	*(Capital cost amount is very important)*	
***THEN***	*(The recycling process is mechanical)*	
***IF***	*(Quality of recovered fibers is very important)*	*AND*
	*(Scalability of the process is not important)*	*AND*
	*(Tolerance for contamination is very important)*	*AND*
	*(Capital cost amount is not important)*	
***THEN***	*(The recycling process is solvolysis)*	

**Table 6:** Quantified values for user criteria.

Recycling Methods	Quality of Recovered Fibers	Scalability and Technology Development Level	Tolerance for Contamination	Capital Costs
Mechanical recycling	1	5	2	5
Pyrolysis	3	4	4	3
Fluidized bed process	3	3	5	2
Supercritical water	5	2	4	1

**Table 7:** Inputs and output provided from the developed system.

**Input Parameters**	**Value**
Weight	1300.0 tons/year
Distance	0.0 km
Type	Prepregs (manufacturing waste)
Recycling process	Pyrolysis
Working capital coefficient	10%
Distribution and selling costs	5%
Number of people	60
Hourly wage	EUR 31.4
**Output**	
Average unit cost per kg of waste	EUR 3.90
Average unit cost per kg of recovered carbon fiber	EUR 6.00

## Data Availability

The relevant data are all included in the paper.
